# Fitness correlates of crop transgene flow into weedy populations: a case study of weedy rice in China and other examples

**DOI:** 10.1111/eva.12377

**Published:** 2016-03-31

**Authors:** Bao‐Rong Lu, Xiao Yang, Norman C. Ellstrand

**Affiliations:** ^1^ Ministry of Education Key Laboratory for Biodiversity and Ecological EngineeringDepartment of Ecology and Evolutionary BiologyFudan UniversityShanghaiChina; ^2^ Department of Botany and Plant SciencesCenter for Conservation BiologyUniversity of CaliforniaRiversideCAUSA; ^3^ Center for Invasive Species ResearchUniversity of CaliforniaRiversideCAUSA

**Keywords:** conspecific weed, evolutionary potential, fitness, herbicide resistance, insect resistance, introgression, *Oryza sativa*, red rice

## Abstract

Whether transgene flow from crops to cross‐compatible weedy relatives will result in negative environmental consequences has been the topic of discussion for decades. An important component of environmental risk assessment depends on whether an introgressed transgene is associated with a fitness change in weedy populations. Several crop‐weed pairs have received experimental attention. Perhaps, the most worrisome example is transgene flow from genetically engineered cultivated rice, a staple for billions globally, to its conspecific weed, weedy rice. China's cultivated/weedy rice system is one of the best experimentally studied systems under field conditions for assessing how the presence of transgenes alters the weed's fitness and the likely impacts of that fitness change. Here, we present the cultivated/weedy rice system as a case study on the consequences of introgressed transgenes in unmanaged populations. The experimental work on this system reveals considerable variation in fitness outcomes ‐ increased, decreased, and none ‐ based on the transgenic trait, its introgressed genomic background, and the environment. A review of similar research from a sample of other crop‐wild pairs suggests such variation is the rule. We conclude such variation in fitness correlates supports the case‐by‐case method of biosafety regulation is sound.

## Introduction

**Figure 1:**
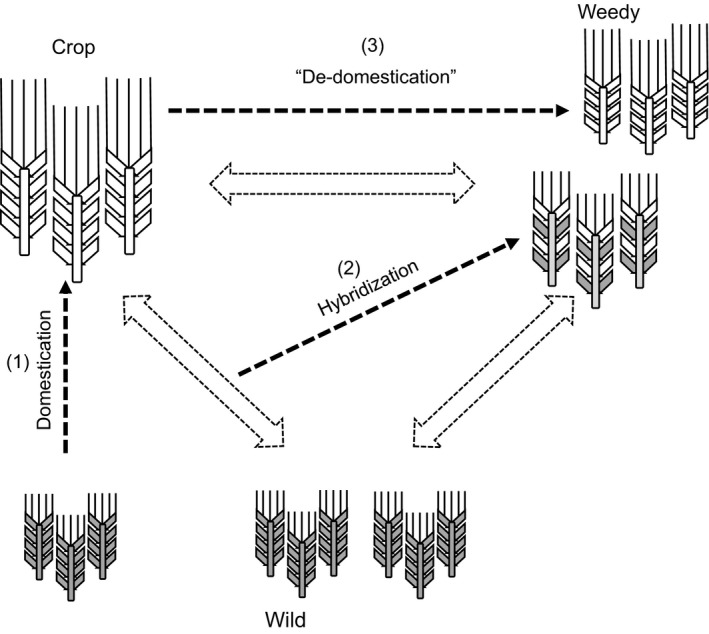
A schematic illustration demonstrating the evolutionary relationships among the populations of a domesticated species (Crop), its wild progenitor (Wild), and a weedy taxon conspecific with the crop (Weedy). Broken arrows indicate historic primary pathways of evolution: Domestication of the crop species (1) from the wild progenitor in the past, and subsequent evolution of the weed populations from natural hybridization (2) between the crop and wild progenitor, and directly through de‐domestication (3) from the crop. Two‐headed arrows indicate continuing sporadic gene flow among the crop, weedy, and wild plants that can result in dynamic evolution of the populations (adapted from Ellstrand 2003a).

Weeds are great challenges for crop production, particularly those that are the same biological species (conspecific) as the crop they infest. For example, weed beets infest sugarbeet fields, and weedy rice infests cultivated rice fields. The phenotypic similarity of such conspecific weeds to the related crops often frustrates visually based hand weeding. Also, genetic similarity means that the crop and the weed are so physiologically similar that herbicide must be applied on the weed with great precision to prevent application on the crop, again requiring visual discrimination (Barrett 1983; Soukup and Holec 2004). Because of their close evolutionary relationship, conspecific weeds are typically cross‐compatible with the related crop species (see Harlan and de Wet 1971; Ellstrand 2003a; Lu 2013). Thus, conspecific weeds represent a unique challenge for their control in crop production because gene flow can deliver useful genes/alleles to weed populations from both their domesticated relatives as well as nearby nonweedy wild relatives (see Fig. 1). This infusion of genetic diversity can provide a substrate for rapid adaptive evolution (Schierenbeck and Ellstrand 2009; Ellstrand et al. 2013). Crop‐to‐weed gene flow has played a significant role in the adaptive evolution of weeds, such as weed beet (Arnaud et al. 2010; Bartsch 2010), weedy rice (Xia et al. 2011a; dos Reis Goulart et al. 2012), and California wild radish (Snow and Campbell 2005). The foregoing examples are a subset of unmanaged populations with introgressed domesticated alleles that have evolved increased weediness or invasiveness (Ellstrand et al. 2013). In addition, crop‐to‐wild gene flow may also affect the evolutionary dynamics of wild populations, causing weed problems (Andersson and de Vincente 2010; Ellstrand et al. 2013).

The advent of genetically engineered (GE) crop species has stimulated discussion about whether crop‐to‐weed and crop‐to‐wild transgene flow might have an ecological or environmental impact (Ellstrand et al. 1999, 2013; Snow et al. 2005; Lu and Yang 2009). Like any other genes, transgenes should move from a GE crop to its non‐GE counterparts and to wild/weedy relatives *via* pollen‐mediated gene flow (Ellstrand 2003b; Stewart et al. 2003; Lu 2008). If a weed/wild population has acquired a transgene that confers a strong selective advantage, and is exposed to a relevant selective pressure (e.g. herbicide spray, pest attacks or drought/salinity stresses), it is likely to exhibit enhanced fitness and evolutionary potential under the natural selection, that may result in unwanted environmental consequences such as increased weediness or invasiveness (Burke and Rieseberg 2003; Ellstrand 2003a; Lu and Snow 2005; Ellstrand et al. 2013). An introgressed transgene with neutral fitness impacts is expected to persist in the population; whereas a transgene with negative fitness impacts is expected to be purged from the population unless is a replenished by subsequent gene flow (Ellstrand 2003a). Thus, a better understanding of correlates of crop transgenes in wild/weedy populations facilitates biosafety assessment of impacts caused by transgene flow. Consequently, the fitness and phenotypic correlates of crop transgene presence under field conditions have been studied in many systems; e.g. squash – wild gourd, maize – teosinte, cultivated sunflower – wild sunflower.

For the world's most important transgenic crops that have been commercialized or are nearing commercialization, the cultivated rice – weedy rice system is perhaps the best studied in that context. In China, a large number of GE rice lines with various transgenes have been developed, and some of them are under biosafety assessment or on their way for commercialization (Huang et al. 2004; Chen and Jia 2008). Spontaneous gene flow is known to occur among domesticated, weedy, and wild rice plants, as in many other crop – wild relative systems (Fig. 1). Therefore, in this article, we use the case of cultivated rice and weedy rice in China to address this topic. We first describe the cultivated rice – weedy rice study system in China. We then present the current state of development of GE cultivated rice for commercialization in China with emphasis on the two traits most likely to be first deregulated: insect resistance and herbicide tolerance. We follow with a review of the research that has been conducted to address the following questions: (i) What is the likelihood of gene flow from cultivated rice to weedy rice? (ii) How does a transgene change the fitness of crop‐weed rice hybrid populations? Finally, we compare the results from rice studies in China with a brief review of a few other systems. Based on these results, we conclude with some of the generalities that have already emerged.

## Weedy rice: origin, genetic diversity and adaptive evolution

Weedy rice, also referred to as “red rice”, is a noxious weed (Holm et al. 1997), extensively infesting rice fields, particularly in Asia, the Americas, Africa, and southern Europe (Delouche et al. 2007; Ziska et al. 2015). Compared to cultivated rice, weedy rice has the following characteristics that contribute to its success as a weed: (i) earlier maturity, (ii) considerable seed shattering; (iii) greater seed persistence in the seed bank; (iv) more robust plant growth, (v) increased reproductive ability; and (vi) greater genetic diversity and phenotypic plasticity (Diarra et al. 1985; Gealy 2005; Delouche et al. 2007). A centuries‐old problem in rice‐growing areas, weedy rice has recently re‐emerged as a problem where direct seeding of cultivated rice has replaced transplanting and less weed control is available (Zhang et al. 2012; Ziska et al. 2015). The presence of weedy rice in cultivated rice fields can cause substantial crop yield losses, varying from 5 to 100%, depending on the weed's density in the field (Gealy 2005; Rao et al. 2007; He et al. 2014). Likewise, when its seeds are coharvested with those of the crop, the result is reduced grain quality (Khush 1997; Lu and Snow 2005). Sometimes, the weed grows so densely that farmers are forced to abandon their rice fields (Abraham and Jose 2014). Thus, weedy rice has become a major threat to rice production and world food security.

The origin and evolution of weedy rice is associated with the domestication of rice, as is the case of the weedy relatives of many other crops (see Fig. 1). Research has revealed that four different evolutionary pathways result in what we call weedy rice *sensu lato* (e.g. Kane and Baack 2007; Londo and Schaal 2007; Xia et al. 2011a; Song et al. 2015; Zhang et al. 2015). First, some populations of weedy rice have directly evolved from wild rice species that grow in habitats that do not depend on human disturbance (e. g. *O. rufipogon* Griff. and *O. nivara* Sharma et Shastry). These wild taxa not only occur as weeds of rice but also as weeds for some other crops (de Wet and Harlan 1975; Harlan 1992). The remaining three pathways result in weedy rice that is restricted to rice fields and their immediate vicinity. Some other weedy rice populations have evolved from natural hybrids between cultivated rice and their reproductively compatible wild relatives (Fig. 1(2), Vaughan et al. 2001; Londo and Schaal 2007). The preceding two pathways involve wild progenitors. Therefore, for these populations, the traits associated with weediness, such as seed dormancy, can be traced back to the wild ancestors.

The third pathway involves hybridization between distantly related rice varieties, such as *indica* and *japonica*, with subsequent recombination. The resulting lines have lost some of their domestication traits and re‐evolved wild phenotypes (such as evolving from nonshattering to shattering). A recent study suggested that the application of hybrid rice may also result in the origin and evolution of some weedy rice populations in China (Zhang et al. 2015). The final pathway, dedomestication (Fig. 1(3)), involves the loss of domestication traits in cultivated rice due to mutation, although not necessarily due to back mutation (e.g. Gross et al. 2010; Xia et al. 2011b), the second type of direct evolution of some weedy rice lines from cultivated rice (Cao et al. 2006; Londo and Schaal 2007; Yao et al. 2015). The latter two evolutionary pathways to weedy rice only involve cultivated progenitors. As a consequence, the genomic constitution of these types of weedy rice is almost exclusively from the crop, with the evolution of a few novel traits not present in the cultivated lines such as red/brown pericarps and temperature‐regulated seed germination, which compensates for the absence of seed dormancy (Gross et al. 2010; Xia et al. 2011b).

Broad definitions of weedy rice involve “plants of the genus *Oryza* that infest and compete with rice and other crops” (Delouche et al. 2007) including plants of wild rice species in the genus *Oryza*. The definition conflates the conspecific weed of human‐managed habitats with wild species that can live and reproduce in both natural and managed habitats. In this article, focus on weedy rice *sensu stricto*, employing Wang et al.'s (2015) definition of weedy rice: “rice plants with strong seed shattering and weediness that only occur inside and in the vicinity of rice fields, and can only reproduce in the human‐disturbed environment.” This definition includes only the three weedy rice lineages that are descended fully or in part from cultivated rice. Thus, weedy rice *sensu stricto* is *Oryza sativa* f. *spontanea*, a free‐living plant that is conspecific with cultivated rice (*Oryza sativa* L.).

The traits of weedy rice relative to its crop ancestor enumerated above enable it reproduce and persist in rice fields for years. The close evolutionary relationship of weedy rice with its cultivated and wild progenitors results in sporadic multiway gene flow in sympatry or peripatry (Fig. 1). Thus, weedy rice populations are often evolutionarily dynamic. They can accumulate genetic diversity as a potential substrate for rapid adaptive evolution. Indeed, studies have shown that weedy rice populations across the world possess abundant phenotypic and genetic diversity (Cao et al. 2006; Kane and Baack 2007; Londo and Schaal 2007; Xia et al. 2011a,b; Zhang et al. 2012; He et al. 2014; Ratnasekera et al. 2014). At one extreme, entire weedy rice populations look superficially similar to wild species or, at the other extreme, nearly identical to cultivars. In general, weedy rice populations vary continuously between the wild and cultivated rice species for their morphological and genetic traits (Vaughan et al. 2001; Ziska et al. 2015), with most weedy rice populations phenotypically favoring their cultivated progenitor.

This trend reflects the adaptive evolution of weedy rice in human‐managed agricultural environments. Pre‐existing variation and that provided by gene flow has enabled the evolution of crop mimicry; that is, weedy rice typically mimics the phenotypic and physiological characters of its locally co‐occurring rice cultivars. Crop mimicry is an evolutionary response to selection under farmers’ visually guided hand weeding (Barrett 1983). Obviously, the evolution of mimicry can be promoted by gene flow and introgression from sympatric cultivated rice (Delouche et al. 2007; Lu and Yang 2009; Xia et al. 2011c). Collectively, crop mimicry and its other characteristics make weedy rice extremely difficult to control once it establishes in a rice field.

Crop‐to‐weed gene flow could provide other traits that might benefit weedy rice populations. In the case of recently improved rice varieties, both transgenic and nontransgenic, these traits include, for example, insect resistance, herbicide tolerance, disease resistance, drought tolerance, salt tolerance, and cold tolerance. As crop gene flow introduces traits into weedy rice populations, the weed can evolve quickly to adapt to the changing crop management practices, such as the shift from seedling transplantation to direct seeding and that from manual and mechanical weeding to weed control by herbicides (Burgos et al. 2008; dos Reis Goulart et al. 2012; Ziska et al. 2015; Merotto et al., this Special Issue). For example, the Clearfield^®^ herbicide‐tolerant rice variety, a product of “mutation breeding” is grown in USA, South and Central Americas, southern Europe, and Southeast Asia (Sudianto et al. 2013). The herbicide/variety system has been successful for weed control in some countries for a short time. But in others, the combination of introgression of the tolerance gene into weedy rice populations and strong selection by the herbicide has rapidly negated the utility of the herbicide/variety weed management system, with severely reduced yields for the farmers using the system (cf., dos Reis Goulart et al. 2012).

What is the future for the evolution of weedy rice under gene flow from GE rice? Whether or not an introgressed transgene will have similar negative consequences will depend on largely four factors: (i) the intended expressed transgene phenotype; (ii) the typical gene flow rates from the crop to the weed; (iii) the phenotype of weed with an introgressed transgene in terms of their ability to reproduce and increase relative to the nonintrogressed weed; and (iv) the introgressed weed's relative ability to reduce the yield of their sympatric crop cultivar. To address these factors, we examine the state of the art in China, is the primary site of GE rice development, for the various intended GE phenotypes being field released and in line for commercialization.

## GE rice in China: research and development

China has invested substantially on developing biotechnology (Huang et al. 2002), including the creation of GE crops. Consequently, a large number of crop varieties/lines with diverse transgenic traits have been produced, some of which have entered commercial production (Chen and Jia 2008). As one of the three most important national crops (rice, wheat and maize), rice is a priority for genetic engineering in China (Chen et al. 2011). During the last two decades, a large number of GE rice lines with diverse transgenic traits have been developed (Table S1). Insect‐resistant and herbicide‐tolerant GE rice lines are prioritized transgenic traits because of the tremendous yield losses caused by insect and weed pests in China's major rice‐growing regions.

The commercialization of insect‐resistant cotton (*Bt*) in China has effectively controlled cotton's major target insect pest, cotton bollworm (*Helicoverpa armigera* Hubner) (Wu et al. 2008, 2009). The success has encouraged scientists developing insect‐resistant rice (Chen et al. 2011). Many GE rice lines have been developed to reduce damage from its primary insect pests (Table S1), especially the chewing lepidopterans (e.g. rice stem borers, leaf‐folders) and sucking hemipterans (e.g., plant‐hoppers and leafhoppers) (Cheng 1996). An important milestone for GE rice was when the Ministry of Agriculture (MOA) of China, authorized by the National Biosafety Committee for Genetically Engineered Organisms, issued two biosafety certificates to two *Bt* rice lines: Huahui‐1 and Bt‐Shanyou‐63 (both lines express a *cry1Ab/Ac* fusion gene) in 2009, after nearly 15 years of assessment for food safety and environmental safety (Lu 2010; see http://www.stee.agri.gov.cn/biosafety/spxx/t20091022_819217.htm). In addition, five other GE lines were also approved for field experiments, including Kemingdao (KMD) (expressing a *cry1Ab* transgene), T1c‐9 (*cry1C* transgene), T2A‐1 (*cry2A* transgene), and Kefeng‐6 and Kefeng‐8 (both expressing tightly linked *cry1Ac*/*CpTI* transgenes) (Chen et al. 2011). The biosafety certificates for Huahui‐1 and Bt‐Shanyou‐63 that were expired in 2014 were renewed by the MOA of China in 2015 for five more years. Chinese rice breeders have produced additional insect‐resistant rice lines by hybridization and back‐crossing between Huahui‐1 and various improved rice varieties.

The development of herbicide‐tolerant GE rice in China has also developed rapidly in the last two decades, motivated by China's recent shift from manual or mechanical weed management to chemical control. As direct seeding rapidly replaces seedling transplantation, highly efficient herbicide‐tolerant GE rice lines are expected to reduce rice production costs. The first herbicide GE rice line containing a *Bar* transgene was developed by the China National Rice Research Institute in the early 1990s (Cao et al. 1992). The *Bar* transgene allows the crop to tolerate the herbicide phosphinothricin (PPT; a. k. a. glufosinate). The transgene expresses the enzyme *phosphinothricin‐Acetyl transferase* which converts PPT into a nonphytotoxic metabolite.

Shortly thereafter, various glyphosate tolerant GE rice lines were developed by transformation with microbial transgenes (e.g. *Agrobacterium* sp. strain CP4) (see Cao et al. 2004). Chinese scientists also developed new types of herbicide‐tolerant transgenes with substantially increased 5‐enolpyruvoylshikimate‐3‐phosphate synthase (*epsps*) expression by adding the strong ubiquitin promotor from maize, or/and the induced mutation *epsps* 102 created via the error‐prone polymerase chain reaction (Su et al. 2008; Wang et al. 2014). The modified *epsps* transgenes were introduced into a widely used rice variety Minghui‐86 via *Agrobacterium* transformation, generating the glyphosate tolerant GE rice line (EP3) (Su et al. 2008).

In the recent years, GE rice lines have been produced with multiple (“stacked”) traits. Transgene stacking can be achieved by various methods such as cotransformation with different transgenes, hybridization between GE lines with different transgenic events, linked transgenes or multigene cassette transformation, and retransformation (Halpin 2005; Naqvi et al. 2009a; Que et al. 2010). Many well‐known GE rice products such as Golden Rice (Naqvi et al. 2009b) contain stacked transgenes. In China, some GE lines containing different stacked insect‐resistance transgenes have been developed to confer wider and stronger resistance to the target insects (see detail in Table S1); other GE rice lines contain a stacked combination of insect‐resistance transgenes with those for other purposes (see detail in Table S1).

By the end of 2010, more than 170 GE rice lines created in China had entered biosafety assessment trials authorized by Chinese government (Zhu 2010). None has yet entered commercial production. Undoubtedly, some GE rice varieties will eventually enter commercial production after the required safety assessments are completed. In particular, a primary source of pressure is some Chinese rice farmers who are enthusiastic about adopting insect‐resistant transgenic rice (Huang et al. 2005). Nonetheless, transgene flow from GE rice to coexisting weedy rice and its consequences remain a concern – and a potential constraint – for the commercialization of GE rice. Therefore, we next examine what is known about gene flow from cultivated rice to its conspecific weed.

## Gene flow: from cultivated to weedy rice

Spontaneous pollen‐mediated gene flow between domesticated crops and spatially adjacent wild/weedy taxa is not uncommon (Ellstrand 2003a,b; Ellstrand et al. 2013). With GE crops growing in field tests and under millions of hectares of commercial cultivation in dozens of countries worldwide, questions of the opportunities for and consequences of transgene flow to wild/weedy have received increasing discussion (Ellstrand 2003a,b; Stewart et al. 2003; Chandler and Dunwell 2008). As mentioned above, one of the major biosafety concerns regarding the field release of GE rice at the scale of commercial production is transgene flow from GE rice varieties to its wild and weedy relatives (Lu et al. 2003; Lu and Snow 2005; Lu and Yang 2009). The primary biosafety concern is that transgenes introgressing into the weedy rice populations that grow intermixed with cultivated rice may lead to unwanted environmental or agronomic consequences (Lu and Snow 2005; Lu and Yang 2009; Chen et al. 2011; Jia et al. 2014).

The extremely close genetic relationship of weedy rice to cultivated rice, their similar phenology, and their high cross‐compatibility suggest that pollen‐mediated gene flow from GE rice to weedy rice populations seems highly probable. This hypothesis is reinforced by the fact that the conspecific weed always occurs within or in near the edge of cultivated rice fields (Delouche et al. 2007; Lu and Yang 2009; Wang et al. 2015). Thus, many studies, descriptive and experimental, have been carried out to measure the extent of gene flow from cultivated rice to weedy rice, both by pollen and by seed. Results from many experimental studies demonstrate that crop‐to‐weed gene flow in rice occurs at a low per‐generation rate (Gealy et al. 2003; Chen et al. 2004; Shivrain et al. 2006; Shivraina et al. 2007; Messeguer et al. 2004; see also Table 1). In general, the estimated frequency of pollen‐mediated gene flow is low most likely due to the predominantly self‐pollination feature and the short life of pollen grains of both cultivated and weedy rice. Low per‐generation gene flow is expected to have significant evolutionary effects when it occurs year‐after‐year (Ellstrand 2003a). Thus, it is not surprising that descriptive studies have revealed crop‐to‐weed gene flow is an important evolutionary force in shaping the genetic diversity and structure of weedy rice populations (Cao et al. 2006; Xia et al. 2011a,b; He et al. 2014; Song et al. 2015).

**Table 1:** Field experiments conducted to detect the frequency of pollen‐mediated (trans)gene flow from cultivated rice to weedy rice

Crop	(Trans)gene	Location	Marker used to detect gene flow	Observed gene flow frequency	References
Glufosinate‐resistant rice	–	United States	Glufosinate‐resistance marker	0.0	Sanders et al. (1998)
Imidazolinone‐resistant rice	–	United States	Imidazolinone‐resistance marker	0.0	Sanders et al. (2000)
Imidazolinone‐resistant rice line ‘CL 2551’	–	United States	Imidazolinone‐resistance marker and SSR molecular finger printing	0.0–0.05%	Estorninos et al. (2002)
GE rice	*gusA* and *bar* gene	Spain	*β*‐glucuronidase marker	0.036 ± 0.006%	Messeguer et al. (2004)
GE rice (Nam29/TR18)	*bar* gene	South Korea	Basta‐resistance marker	0.011–0.046%	Chen et al. (2004)
Imidazolinone‐resistant Clearfield^™^ (CL) rice	–	United States	Imidazolinone‐resistance marker and SSR molecular finger printing	0.003–0.008%	Shivraina et al. (2007)
GE rice	PPT‐R	Costa Rica	Glufosinate‐resistance marker	1.0–2.3%	Olguin et al. (2009)
GE rice	*Protox* (protopor‐phyrinogen oxidase) gene	South Korea	PPO‐resistance marker	0.039%	Chun et al. (2011)
GE rice	*bar* gene	China	Basta‐resistance marker	0.002–0.342% and 0.090%	Jia et al. (2014)
*Indica* and tropical *japonica* rice cultivars	–	United States	SSR molecular finger printing	0.0	Gealy et al. (2015)
GE rice (Xiang 125S/Bar68‐1)	*Bar* gene	China	Glufosinate‐resistance marker	0.395–0.470% and 0–0.187%	Sun et al. (2015)

“–” indicates a nontransgenic variety.

Pollen‐mediated gene flow can occur in both directions, namely, crop‐to‐weed and weed‐to‐crop. But unless some of the harvested grain is replanted, the important maternal source of weedy hybrids will be the weeds. If GE rice is involved in the crop‐weed gene flow and introgression, the resulted weedy hybrid progeny will contain one or more transgenes. Such recurrent gene flow and subsequent introgression will play roles in the evolutionary dynamics of the weedy populations. Commonly, antibiotic (e.g. hygromycin resistance, *hpt*) and herbicide tolerance genes are used as selectable markers in GE rice transformation. They also have been utilized as markers in various experiments to detect the frequencies of rice transgene flow (Chen et al. 2004; Rong et al. 2005, 2007). As shown in Table 1, molecular markers (e.g. microsatellites) are also used to quantify of crop‐to‐weed and crop‐to‐wild gene flow (Messeguer et al. 2004; Olguin et al. 2009; Chun et al. 2011; Jia et al. 2014). Gene flow results collected from these experimental studies are the first step for assessing the impact of crop‐to‐weed and crop‐to‐wild transgene flow in rice.

The earliest *Oryza* crop‐to‐weed gene flow field experiments are from Sanders et al. (1998, 2000) who used two herbicide‐tolerant rice varieties (to imidazolinone‐ and glufosinate) in independent studies at the Louisiana State University Research Farm in the United States. In these experiments, weedy rice (referred as “red rice” in their study) populations were interplanted with the cultivated rice varieties. The gene flow frequencies were estimated by screening seedlings from weedy rice parents with the appropriate herbicide; crop‐weed hybrid seedlings would be expected to survive. Extremely few seedlings from weedy rice parents interplanted with the glufosinate‐tolerant rice survived glufosinate screening. The few weedy rice plants surviving from the imidazolinone spray were found NOT to be products of natural hybridization. Thus, the researchers estimated that gene flow was extremely low (close to zero) (Sanders et al. 1998, 2000). Soon after, Estorninos et al. (2002) used microsatellite markers to determine the outcrossing rate between the imidazolinone‐tolerant rice line “CL 2551” and awnless straw‐hulled weedy rice at Stuttgart, Arkansas, USA; the outcrossing rate was estimated to be 0.0–0.05%. After reviewing more than ten published studies, Gealy et al. (2003) concluded that rice crop‐to‐weed gene flow was extremely low (<1%) but highly variable. Later field experiments supported that conclusion. For example, gene flow frequencies from glufosinate‐tolerant GE rice to nearby several weedy rice accessions ranged from 0.0 to 0.5% (Chen et al. 2004). Likewise, Shivraina et al. (2007) reported a comparable amount of crop‐to‐weed gene flow in a field experiment that allowed for measuring much greater spatial scale between source and sink plants; frequencies of 0.003–0.008% crop‐to‐weed gene flow were recorded. Using a similar transgene marker, Jia et al. (2014) investigated the frequency of gene flow from the crop into two weedy rice populations: one in Leizhou of Guangdong province, the other in Yangzhong of Jiangsu province. The estimated gene flow ranged from 0.002% to 0.342%. Sun et al. (2015) assessed pollen‐mediated gene flow from glufosinate‐resistant (GR) transgenic hybrid rice to six weedy rice accessions, and found that the frequency of gene flow from GR rice to weedy rice accessions ranged from 0% to 0.47% in different designs of gene flow experimental. To date, the highest frequency of crop‐to‐weed gene flow was reported by Olguin et al. (2009) who studied the transgene flow from *indica* rice to 58 weedy rice accessions from Costa Rica; it was as high as 2.3%. Notably, Pu et al. (2014) also reported insect‐mediated pollination in rice, where increased frequency of transgene flow was detected.

As a group, the above studies indicate that – although the gene flow frequency per generation is very low – it also varies considerably under different situations. Given that some gene flow is almost always present when the crop and the weed co‐occur and that it will be recurrent if rice is planted in the same location every year, transgene flow and eventual introgression from a GE rice variety to weedy rice populations seems inevitable. It is probably impossible to stop the flow of GE rice transgenes into weedy rice populations without extraordinary efforts (NAS 2004).

But whether transgene introgression will lead to increased weed problems and/or environmental impacts also depends on any fitness or other evolutionarily significant phenotypic changes in weedy rice individuals that are associated with the introgressed transgene(s) (Lu and Yang 2009; Ellstrand et al. 2014). If gene flow is expected to occur, then such data are next critical step in environmental risk assessment associated with transgene flow (e.g. Hokanson et al. 2010, 2015; Huesing et al. 2011). Thus, the following questions need to be considered prior to commercialization of GE where weedy rice is present: What will be the agronomic and ecological consequences of introgressed transgenes in a weedy rice population. Will an introgressed transgene change the fitness and evolutionary dynamics of a weedy population?

## Fitness and evolutionary dynamics: field performance of transgenic crop‐weed hybrid progeny and their descendants

**Figure 2:**
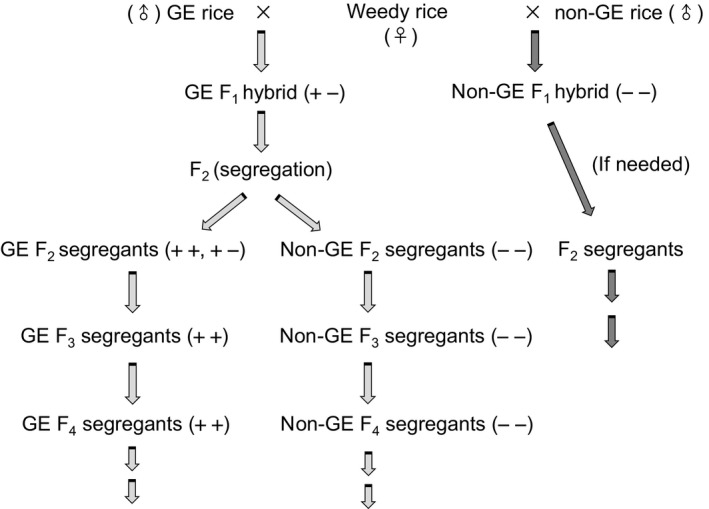
A schematic illustration showing the establishment of crop‐weed rice hybrid lineages with or without a transgene for the comparison of fitness effects between the transgene‐present and transgene‐absent individuals in different generations. ++, homozygous transgenic hybrid lineages/segregants; +−, heterozygous transgenic hybrid lineages/segregants; − −, nontransgenic hybrid lineages/segregants.

Measuring the fitness effect of a crop transgene in individuals with a history of crop‐weedy/wild hybridization requires one or more field experiments that compare wild/weedy individuals in which the transgene is present and with those in which the transgene is absent. Such individuals can be created with artificial crosses of a transgenic crop line and its nontransgenic counterpart (the line that was transformed to create the transgenic line) with their weedy (or wild) relatives as illustrated in Fig. 2. Measurement of the comparative performance of the two types of hybrid progeny or their descendants in the field – such as the survival ratios, competitiveness, and fecundity under specific environmental conditions – reveals the fitness effect to the wild/weedy relative populations associated with the presence of the transgene (Lu and Yang 2009).

The fitness effects of a transgene are expected to be largely determined by the type of transgenes (e.g. insect resistance and herbicide resistance) incorporated in wild/weedy population the type of anticipated selection pressure in the environment, and the amount of admixture in the transgenic hybrid lineages (Lu and Yang 2009; Yang et al. 2012, 2015; Xia et al. 2016). Therefore, three types of characteristics should be carefully used when estimating the potential short‐term evolutionary dynamics and long‐term ecological impacts of a transgene that has been incorporated in weedy/wild populations: (i) possible positive or negative fitness changes associated with a particular transgene when any intended selective pressure is absent (e.g. plants bearing a virus‐resistant transgene in the absence of the specific virus); (ii) fitness changes under the intended selective pressure that is related to a particular transgenic trait in the intended specific environment; and (iii) fitness effects of a transgene in early and advanced generations of the transgenic hybrid lineage compared with their nontransgenic counterparts, including the pure wild/weedy ancestral types. This approach is in accord with the case‐by‐case principle for biosafety assessment of genetically engineered crops (NAS 2002).

We have conducted multiyear common‐garden experiments to estimate the fitness effect of insect‐resistance transgenes that transformed into cultivated rice (Chen et al. 2006; Xia et al. 2010, 2011c) and introgressed into weedy (Cao et al. 2009; Yang et al. 2011, 2012, 2015; Zhang et al. 2013; Xia et al. 2016) and wild rice populations (Dong et al. 2011) to understand their potential impact. Regarding transgene flow to weedy rice, one of our major foci, we used a number of GE rice lines containing different insect‐resistance transgenes (*Bt*,* CpTI*,* Bt/CpTI*) to cross with weedy rice populations from different geographic locations. *Bt* and *CpTI* transgenic rice lines were developed to deter lepidopteran pests, such as rice stem borers and rice leaf‐folders. Several generations of crop‐weed hybrid progeny (F_1_–F_7_) with transgene‐present and transgene‐absent lineages were produced to study the long‐term fitness effect of the insect‐resistance transgenes. We compared an array of fitness‐related traits (such as plant height, number of tillers, number of panicles, and number of well‐developed seeds) for these hybrid lineages, as well as their crop and weedy progenitor under both natural‐insect and low‐insect pressure. The multiyear experiment found the following general trends:

1. Insect‐resistance transgenes are effective and stable in terms of their resistance to target insects in crop‐weed hybrid progeny and their descendants. When targeted insects were abundant in the field, they provide a stable fitness benefit to transgenic hybrid‐descended lineages in terms of more tillers, panicles, and well‐developed seeds, However, the insect‐resistance transgenes provide no or negligible fitness benefit to the transgene‐present hybrid lineages when target insects are absent or occur at extremely low frequencies (Yang et al. 2011, 2012, 2015).
2. Ambient insect pressure as mediated by the local genetic composition of crop‐weed hybrid derived populations also plays an important role in determining the fitness effect of the insect‐resistance transgenes (Yang et al. 2012; Xia et al. 2016). Insect pressure is significantly reduced in the field experimental plots where the transgenic and nontransgenic hybrid plants are mixed alternately. Apparently, the presence of insect‐resistant plants at that frequency and spatial distribution is sufficient to extend any advantage to adjacent nontransgenic plants such that no significant difference in fitness occurs (Yang et al. 2012, 2015).
3. Taking our results collectively, we conclude that introgression of insect‐resistance transgenes from GE rice into coexisting weedy rice populations is unlikely to cause increased weediness or other environmental problems. The explanation is that the generally reduced insect infestation in rice ecosystems by the extensive cultivation of insect‐resistant GE rice will bring no relative fitness benefit to the sympatric weedy rice populations, whether they receive the transgene or not (Yang et al. 2012). The significantly reduction of target insects observed in cotton fields after the extensive and long‐term commercial cultivation of transgenic *Bt* cotton (Wu et al. 2008) backs up our conclusion that the presence of extensive insect resistance will result in the substantial decrease in targeted insect pests of rice.

With regard to herbicide‐resistance transgenes, Oard et al. (2000) conducted a field experiment and evaluated the seed production, shattering, and dormancy in eight F_2_ populations produced from controlled crosses of two transgenic, glufosinate‐resistant rice lines and four red rice biotypes. The presence of the transgene was associated with significantly shorter plant height and different maturity in hybrids, compared to those in nontransgenic counterparts. In another study, Wang et al. (2014) carried out a multiple‐year common garden study on the fitness effects of a transgene that confers tolerance to the herbicide glyphosate through overexpression of 5‐enolpyruvoylshikimate‐3‐phosphate synthase (*epsps*). In a glyphosate‐free environment, they found that the transgenic hybrid lineage had higher seed production, greater EPSPS protein levels, tryptophan concentrations, photosynthetic rates, and percent seed germination compared with nontransgenic controls. These results suggest that phenotypic changes associated with such an *epsps* transgene could result in faster establishment and increased competitive ability of the transgene‐bearing individuals compared to nontransgenic weedy rice and cultivated rice.

Other field studies of weedy rice individuals with introgressed non‐GE crop herbicide resistance have shown fitness advantages related with faster germination (Goulart et al. 2012) and taller plants (Shivrain et al. 2006). Gene exchange and the spread of herbicide‐resistant alleles in weedy rice suggest that wide‐scale adoption of transgenic herbicide‐resistance rice as a means to control weedy rice will be limited unless biotechnological solutions could be used to significantly decrease the occurrence of gene flow (NAS 2004) or otherwise mitigate it (Gressel 1999).

The next of generation GE rice in China is apt to involve multiple transgenes with different intended purposes (pyramided and stacked) traits to battle the complex of insect pests of rice and other yield constraints in rice (Chen et al. 2011). Such products of crop biotechnology will inevitably require even more complicated experimental approaches for environmental biosafety assessment with regard to introgressed transgenes in weedy rice populations. More biotic and/or abiotic factors will have to be taken into account to simulate field conditions that may influence the fitness effect of the multiple transgenes employed simultaneously.

The environmental biosafety assessment of the consequences of crop transgene flow to weedy rice populations through artificially created crop‐weed hybrid lineages and subsequent fitness testing has proven costly and time consuming (Jenczewski et al. 2003; Lu and Yang 2009), especially with regard to long‐term evolutionary impacts. However, our multiple‐year field experiment on insect‐resistant transgenic crop‐weed hybrid progeny reveal that the experimental estimation of fitness effects should be sufficiently apparent based on data from hybrid lineage as early as two or three generations posthybridization (Yang et al. 2015). Also, it should be sufficient to make some preliminary conclusions on the impacts caused by the insect‐ and herbicide‐resistance transgene flow into weedy rice population case‐by‐case, based on results obtained from above studies. (i) For the insect‐resistance transgene, the fitness advantages brought by insect‐resistance transgene might be limited due to the fact that weedy plants will be surrounded by insect‐resistant plants in a GE rice field, and the extensive commercial cultivation of an insect‐resistant GE crop will largely reduce target herbivores in a GE deployed area as previously described by Wu et al. (2008) for *Bt* cotton. As a result, insect‐resistance transgenes flow into weedy rice will have a limited evolutionary impact. (ii) In contrast, herbicide‐resistance transgenes should deserve more attention since the herbicide pressure in GE rice field will always favor the spread and fixation of any herbicide‐resistance transgene in weedy rice population, unless effective mitigation strategy to be developed to minimize the occurrence of gene flow or reduce the fitness of crop‐weed hybrids.

## Conclusions

### Weedy rice in China

Weedy rice is a noxious weed that causes tremendous yield losses for cultivated rice worldwide. The control of weedy rice is challenging because of its unique characteristics, such as strong seed shattering and dormancy, abundant genetic diversity, and mimicry with cultivated rice varieties. Like other conspecific weeds, weedy rice can easily acquire genes/alleles from its cultivated progenitors through crop‐to‐weed gene flow. Some crop alleles can also enhance the fitness of weedy rice, enabling it to adapt to and evolve rapidly in the cultivated rice agro‐ecosystem. Although Crop‐to‐weed (trans)gene flow in rice occurs at a relatively low frequency (0.001–2.3% per generation), transgene introgression into weedy population is essentially inevitable because weedy rice and cultivated rice co‐occur and year‐after‐year in the same fields. Transgene flow from GE rice to weedy rice can result in diverse fitness effects, depending on the type of transgenes and the selective pressure to which the GE crop‐weed hybrid descendants are exposed. In addition, other factors such as the genetic background of weedy rice populations that have obtained the transgenes may also influence the fitness effects of a particular transgene (Xia et al. 2016).

Many studies have indicated that insect‐resistance transgenes (e.g. *Bt* and *Bt*/*CpTI*) confer a fitness benefit for crop‐weed rice hybrid progeny under high insect pressure (Cao et al. 2009; Yang et al. 2011, 2012, 2015; Xia et al. 2016). However, the same transgenes do not confer such a benefit to the hybrid progeny under low‐insect pressure. The fitness studies based on multiple generation descendants of GE crop‐weed hybrids provide similar results (Yang et al. 2011, 2012, 2015). Therefore, we conclude that fitness change and evolutionary potential for transgene flow from GE insect‐resistant rice to weedy rice populations are quite limited because of the low ambient insect pressure expected in extensively planted transgenic commercial rice production fields (Yang et al. 2012; Xia et al. 2016). In contrast, the movement of herbicide‐resistance transgene (*EPSPS*) to weedy rice populations appears to considerably change the fitness of the crop‐weed hybrid progeny, both with and without the application of glyphosate herbicide sprays, and possibly the evolutionary potential of the hybrid progeny by altering their rate of biosynthesis and photosynthesis (Wang et al. 2014). This indicates that the movement of this specific herbicide‐resistance transgene (event) to weedy rice populations may result in increased weed problems.

### Fitness correlates of introgressed transgenes

Our understanding of the fitness effects and expected evolutionary dynamics brought by transgenes, including those conferring herbicide resistance, drought and cold tolerance, and stacked traits with diverse functions are still limited. It is clearly shown from the results of studies already done on the cultivated rice and weedy rice system that simple expectations from the transgene's intended phenotype are not sufficient to predict what will occur under experimental conditions.

Are these results general? We sought to compare the results discussed above with a sample of results from similar field experiments involving different species and/or different transgenes. We review a collection of such studies that are included in Table 2. Our sample involves twelve studies representing six crop donor species and eight recipient weedy/wild species. Four different transgenic phenotype classes are represented. The only generality that emerges is variability. Introgressed transgenes may or may not confer a fitness advantage under selective pressure associated with the intended transgenic phenotype. Without that selective pressure, the presence of the transgene may correlate with increased fitness, decreased fitness, or no significant fitness change. Taken collectively, the cultivated rice – weedy rice system case study reviewed above and the additional studies featured in Table 2 make it clear that the fitness changes associated with transgenic presence in unmanaged populations cannot be predicted *a priori*. While increased fitness in itself may not be sufficient to predict an environmental hazard, it does provide support for the conclusion that the transgene will persist and spread (Ellstrand 2003a). Obviously, with regard to introgression‐based transgene risk assessment, the current regulatory policy of case‐by‐case analyses informed by field‐based research is sound and superior to predicting the fitness correlates of introgressed transgenes without such data.

**Table 2:** Examples of fitness correlates of crop transgenes in partially introgressed nondomesticated genomes under experimental field conditions

Crop taxon	Wild/weedy taxon	Trait of transgene(s)	Generation compared	Fitness correlates of transgene: presence vs absence		References
				Under selective pressure related to intended transgene phenotype	Without such selective pressure	
*Brassica napus*, oilseed rape	*B. rapa,* wild bird's rape	Herbicide (glyphosate) resistance	BC_1_	More seeds produced	Fewer seeds produced	Londo et al. (2010)
*Curcurbita pepo*, crookneck squash	*C. pepo* ssp. *texana*, wild gourd	Virus disease resistance (for 3 different viruses)	F_1_, BC_1_‐BC_4_	More flowers and fruits produced; but more damage by beetles and bacterial infection	None	Sasu et al. (2009, 2010)
*Glycine max*, soybean	*G. soja*, wild soybean	Herbicide (glyphosate) resistance	F_2_	Not studied	None	Guan et al. (2015)
*Helianthus annuus*, sunflower	*H. annuus*, wild sunflower	Fungal (white mold) disease resistance	F_1_	None	None	Burke and Rieseberg (2003)
*Oryza sativa*, rice	*O. sativa* f. *spontanea*, weedy rice	Herbicide (glyphosate) resistance	F_1_ and F_2_	Not studied	More seeds produced	Case study reviewed in this article
*Zea mays mays*, maize, corn	*Z. mays* ssp. *mexicana,* teosinte	Herbicide (glyphosate) resistance	F_1_	Not studied	None	Guadagnuolo et al. (2006)
*Oryza sativa*, rice	*O. sativa*. f. *spontanea*, weedy rice	Insect (*lepidoptera*) resistance	F_2_–F_7_	More seeds produced	None	Case study reviewed in this article
*Oryza sativa*, rice	*O. rufipogon*, wild rice	Insect (*lepidoptera*) resistance	F_2_–F_4_, BC_1_, BC_1_F_2_ and BC_1_F_3_	Not studied	None	Dong et al. (2011)
*H. annuus*, sunflower	*H. annuus*, wild sunflower	Insect (*lepidoptera*) resistance	BC_1_	More seeds produced	None	Snow et al. (2003)
*B. napus*, oilseed rape	*B. rapa,* wild bird's rape	Insect (*lepidoptera*) resistance	F_1_, BC_1_F_1_, BC_2_F_1_ and BC_2_F_2_	Not studied	None	Halfhill et al. (2005)
*B. napus*, oilseed rape	*B. rapa,* weed bird's rape	Insect (*lepidoptera*) resistance	F_1_	More seeds and biomass produced	None	Sagers et al. (2015)
*B. napus*, oilseed rape	*B. juncea*, wild bird's rape	Insect (*lepidoptera*) resistance	BC_2_	More seeds produced	More seeds produced	Liu et al. (2014)

## Supporting information


**Table S1**. Transgenic traits successfully introduced into cultivated rice (*Oryza sativa*) through genetic engineering in China.
