# Supplementary material for: Fitness correlates of crop transgene flow into weedy populations: a case study of weedy rice in China and other examples
Source: Evol Appl. 2016 Mar 31;9(7):857–70. doi: 10.1111/eva.12377 (PMC4947148; doi:10.1111/eva.12377)
Supplement: Supplementary file 1 — Table S1. Transgenic traits successfully introduced into cultivated rice (Oryza sativa) through genetic engineering in China. [file EVA-9-857-s001.doc]

Table S1. Transgenic traits successfully introduced into cultivated rice (*Oryza sativa*) through genetic engineering in China.

| Trait | Gene | Donor | Transgenic product | Function | References/Codes |
| --- | --- | --- | --- | --- | --- |
| Insect resistance | | | | | |
|  | *pinII* | Potato | Potato proteinase inhibitor II (pinII) | Inhibit trypsin and chymotrypsin simultaneously | Duan et al. 1996 |
|  | *GNA* | Snowdrop | *Galanthus nivalis* agglutinin (GNA) | Specificity to α-1,3- or 1,6-linked d-mannose residues in carbohydrates | Rao et al. 1998 |
|  | *Bt aizawai* 7-  29* | *Bacillus thuringiensis* | δ-endotoxin protein gene | Once activated, the endotoxin binds to the gut epithelium and causes cell lysis by the formation of cation-selective channels | Yang et al. 1989;  Xie et al. 1991 |
|  | *CpTI* | Cowpea | Cowpea trypsin inhibitor | Exhibit a strong inhibitory activity against trypsin | Xu et al. 1996 |
|  | *Cry1Ab* | *B. thuringiensis* | Cry1Ab toxic protein | Bind reversibly to receptors on the surfaces of larval midgut and form ion channels | Shu et al. 2000;  Wang et al. 2014 |
|  | *Cry1Ac* | *B. thuringiensis* | Cry1Ac toxic protein | Same as above | Xiang et al. 1999; Zeng et al. 2002;  Chen et al. 2008 |
|  | *Cry1C* | *B. thuringiensis* | Cry1C toxic protein | Same as above | Tang et al. 2006  Ye et al. 2009 |
|  | *Cry2A* | *B. thuringiensis* | Cry2A toxic protein | Same as above | Chen et al. 2005 |
|  | *Cry9C* | *B. thuringiensis* | Cry9A toxic protein | Same as above | Chen et al. 2008 |
|  | *Cry1Ab/Cry1Ac* | *B. thuringiensis* | Cry1Ab and Cry1Ac toxic protein | Same as above | Liu et al. 2012 |
|  | *Cry1Ab/Ac* | *B. thuringiensis* | Cry1Ab/Ac toxic protein | Same as above | Tu et al. 2000;  Ye et al. 2001 |
|  | *Cry1Ac+Sck* | *B. thuringiensis*  Cowpea | Cry1Ac toxin protein  Cowpea trypsin inhibitor | Cry1Ac binds reversibly to receptors on the surfaces of larval midgut and form ion channels; CpTI exhibit a strong inhibitory activity against trypsin | Zhao et al. 2004 |
|  | *spI^e^* | Spider | Insecticidal toxins | Conferring resistance to leaf-folder and striped stem borer | Huang et al. 2001 |
| Disease resistance | | | | | |
|  | *Pib* | Rice |  | Enhance rice blast disease resistance | Wang et al. 1999 |
|  | *Pi-ta* | Rice |  | Enhance rice blast disease resistance | Bryan et al. 2000 |
|  | *CP,SP* | Rice stripe virus | Coat protein (CP), special-disease protein (SP) and chimeric CP/SP gene sequence | Preventing rice stripe disease by RNA interference | Ma et al. 2011 |
|  | *CP* | Potato virus Y | Coat protein | Preventing rice stripe disease by RNA interference | Zhang et al. 2012 |
|  | *Xa21* | *Oryza longistaminata* | Receptor kinase-like protein | Enhance resistance to bacterial blight | Song et al. 1995 |
|  | *pemG1* | *Magnaporthe grisea* | Phenylalanine ammonia-lyase | *PemG1* is elicitor-encoding gene that stimulate defense responses in plants | Qiu et al. 2009 |
| Yield increase | | | | | |
|  | *Tzs, ipt* | A. tumefaciens | Isopentanyl transferase (IPT) | Stunted growth, loss of apical dominance, delayed senescence, and reduced root formation | Cao et al. 2004 |
|  | *VHB* | Vitreoscilla | Vitreoscilla hemoglobin (VH) | VH facilitates oxygen transfer to the respiratory membranes | Cao et al. 2004 |
|  | *yld1.1, yld2.1* | Rice |  |  |  |
|  | OsPHF1 | Rice | Phosphate transporter traffic facilitator1 | Regulate the plasma membrane localization of low- and high-affinity inorganic phosphate transporters and determines inorganic phosphate uptake and translocation | Chen et al. 2011 |
|  | *LRK1* | *Oryza rufipogon* Griff. | Leucine-rich repeat receptor-like kinase | Regulate rice branch number by enhancing cellular proliferation | Zha et al. 2009 |
|  | *PEPC, PPDK* | Maize | Phosphoenolpyruvate carboxylase,  Pyruvate orthophosphate dikinase | Enhance the photosynthetic capacity of transgenic rice with C4 gene | Zhang et al. 2009 |
|  | *Ghd8* | Rice | HAP complex | Ghd8 up-regulated MOC1, a key gene controlling tillering and branching; this increased the number of tillers, primary and secondary branches, thus producing 50% more grains per plant. | Yan et al. 2011 |
|  | | | | | |
| Quality and nutrient efficiency improvement | | | | | |
|  | *Psy,Crt1* | *Narcissus pseudonarcissus,*  *Erwinia uredovora* | Phytoene synthase and Phytoene  dehydrogenase | Produce beta-carotene, a precursor of Vitamin A | Ye et al. 2000 |
|  | *OsNPF2. 4* | Rice | Nitrate transporter gene | Improved growth and nitrogen use efficiency expressing nitrate transporter gene | Xia et al. 2015 |
|  | *Wx* | Rice | *Antisense Waxy (Wx) gene* | Reduce amylose synthesis | Liu et al. 2003 |
|  | *SUSIBA2* | Barley | Sugar signalling in barley 2 | Increased starch content and reduce methane emissions | Su et al. 2015 |
| Salt tolerance | | | | | |
|  | *BADH* | Sugar Beet | Betaine aldehyde dehydrogenase (BADH) | Synthesis of glycinebetaine into salt-sensitive crops leading to accumulation of glycinebetaine and improvement of salt tolerance | Guo et al. 1997 |
|  | *mtID, gutD* | *Escherichia coli* | Mannitol-1-phosphate dehydrogenase (mtID), Glucitol-6-phosphate dehydrogenase(gutD) | Produce and accumulate mannitol to increase the osmotic pressure, improve salt tolerance | Wang et al. 1999 |
|  | *SsNHX1* | *Suaeda salsa* | Suaeda salsa vacuolar Na+/H+ antiporter | Removal of Na+ from the cytoplasm by transporting it into vacuole via Na+/H+ exchangers | Zhao et al. 2006 |
|  | *OsJAZ9* | Rice | Plant-specific TIFY proteins | *OsJAZ9* acts as a transcriptional regulator by forming a transcriptional regulation complex with *OsNINJA* and *OsbHLH* to fine tune the expression of JA-responsive genes involved in salt stress tolerance | Wu et al. 2015 |
|  | *OsCIPK03* | Rice | Calcium sensor-interacting protein kinase | *OsCIPK03* functions as a negative regulator of salt stress tolerance in rice | Rao et al. 2011 |
| Drought tolerance | | | | | |
|  | *SNAC1* | Rice |  | Delayed leaf-rolling and reduced rate of water loss | Hu et al. 2006 |
|  | *MnSOD* | Pea | Manganese superoxide dismutase (MnSOD) | MnSOD evolved mechanisms to scavenge reactive oxygen species | Wang et al. 2005 |
|  | *hrf1* | Rice | Harpin proteins | Overexpression of a Harpin-encoding gene hrf1 in rice increased drought tolerance through abscisic acid (ABA) signalling. | Zhang et al. 2011 |
|  | *ZFP245* | Rice | Zinc finger protein | ZFP245 may contribute to the tolerance of rice plants to cold and drought stresses by regulating proline levels and reactive oxygen species-scavenging activities | Huang et al. 2009 |
|  | *JERF3* | Rice | Ethylene response factor (ERF) proteins | Overexpression of JERF3 increases contents of soluble sugars and proline under dehydration conditions; leds to the up-regulated expression of two OsP5CS genes in response to drought treatment ; activates the expression of stress-responsive genes, including WCOR413-like, OsEnol, and OsSPDS2 | Zhang et al. 2010 |
|  | *OsTPS1* | Rice | Trehalose | Overexpression of OsTPS1 may enhance the abiotic stress tolerance of plants by increasing the amount of trehalose and proline, and regulating the expression of stress-related genes | Li et al. 2011 |
|  | *OsSDIR1* | Rice | E3 ligase | OsSDIR1 could complement the drought sensitive phenotype of the sdir1 mutant were more sensitive to ABA | Gao et al. 2011 |
|  | | | | | |
| Herbicide resistance | | | | | |
|  | *Bar* | *Streptomyces hygroscopicus* | *Phosphinothricin-Acetyl transferase* | Converts phosphinothricin (PPT) to a nonphytotoxic metabolite | Cao et al. 1992 |
|  | *epsps* | *Agrobacterium* sp. strain CP4 | 5-enolpyruvylshikimate-3-phosphate synthase | Enhance Glyphosate resistance | Cao et al. 2004 |
|  | *epsps*  *epsps102* | Rice | 5-enolpyruvylshikimate-3-phosphate synthase | Enhance Glyphosate resistance | Zhou et al. 2006;  Su et al. 2008 |
|  | | | | | |
| Stacked traits | | | | | |
|  | *Cry1Aa+pta^f^* | *B. thuringiensis*  *Pinellia ternate* agglutinin | Cry1Aa toxin protein  *Pinellia ternate* agglutinin | Broad spectrum resistance to homopteran (sap-sucking), Lepidopteran insects | Lin et al. 2006 |
|  | *Cry1Ab/Ac+Xa21* | *B. thuringiensis*  *O. longistaminata* | Cry1Ab /Ac toxin protein  Receptor kinase-like protein | Cry1Ab/Ac binds reversibly to receptors on the surfaces of larval midgut and form ion channels; Xa21 enhance resistance to bacterial blight | Jiang et al. 2004 |
|  | *Gna+sbtig* | Snowdrop  Soybean | Galanthus nivalis agglutinin  Soybean trypsin inhibitor | Gna has specificity to α-1,3- or 1,6-linked d-mannose residues in carbohydrates, is toxic to insects; Sbtig exhibits a strong inhibitory activity against trypsin ; Resistance to homopteran (sap-sucking), Lepidopteran insects | Li et al. 2003 |
|  | *Gna+Xa21* | Snowdrop  *O. longistaminata* | Galanthus nivalis agglutinin  Receptor kinase-like protein | Gna has specificity to α-1,3- or 1,6-linked d-mannose residues in carbohydrates, is toxic to insects;  Xa21 Enhances resistance to bacterial blight | Tang et al. 1999 |
|  | *Cry1Ab+Bar+Xa21* | *B. thuringiensis*  Streptomyces hygroscopicus  *O. longistaminata* | Cry1Ab toxin protein  phosphinothricin-Acetyl transferase  Receptor kinase-like protein | Cry1Ab binds reversibly to receptors on the surfaces of larval midgut and form ion channels; Bar converts phosphinothricin (PPT) to a nonphytotoxic metabolite; Xa21 enhances resistance to bacterial blight | Wang et al. 2002 |
|  | *Cry1Ab+ pinII+Bar* | *B. thuringiensis*  Potato  *O. longistaminata* | Cry1Ab toxin protein  Potato proteinase inhibitor II  Receptor kinase-like protein | Binds reversibly to receptors on the surfaces of larval midgut and form ion channels; | Zhu et al. 1999  Yao et al. 2002 |
|  | *CryAb+vip3H* | *B. thuringiensis* | CryAb toxin protein  Vip3 toxins | Resistance to homopteran (sap-sucking), Lepidopteran insects | Fang 2008  Chen et al. 2010 |

Reference for Table S1

Cao, J., X. L. Duan, D. McElroy, and R. Wu. 1992. Regeneration of herbicide resistant transgenic rice plants following microprojectile-mediated transformation of suspension-culture cells. Plant Cell Reports **11**:586–591.

Cao, M. X., J. Q. Huang, Z. M. Wei, Q. H. Yao, C. Z. Wan, and J. A. Lu. 2004. Engineering higher yield and herbicide resistance in rice by Agrobacterium-mediated multiple gene transformation. Crop Science **44**:2206–2213.

Chen, H., G. Zhang, Q. Zhang, and Y. Lin. 2008. Effect of transgenic *Bacillus thuringiensis* rice lines on mortality and feeding behavior of rice stem borers (Lepidoptera: Crambidae). Journal of Economic Entomology **101**:182–189.

Chen, H., W. Tang, C. G. Xu, X. H. Li, Y. J. Lin, and Q. F. Zhang. 2005. Transgenic indica rice plants harboring a synthetic *cry2A** gene of *Bacillus thuringiensis* exhibit enhanced resistance against lepidopteran rice pests. Theoretical and Applied Genetics **111**:1330–1337.

Chen, Y., J. C. Tian, Z. C. Shen, Y. F. Peng, C. Hu, Y. Y. Guo, and G. Y. Ye. 2010. Transgenic rice plants expressing a fused protein of Cry1Ab/Vip3H has resistance to rice stem borers under laboratory and field conditions. Journal of Economic Entomology **103**:1444–1453.

Chen, J., Y. Liu, J. Ni, Y. Wang, Y. Bai, J. Shi, J. Gan et al*.* 2011. *OsPHF1* regulates the plasma membrane localization of low-and high-affinity inorganic phosphate transporters and determines inorganic phosphate uptake and translocation in rice. Plant Physiology **157**: 269–278.

Chen, S., D. Ni, X. Lu, L. Li, W. Wang, Q. Zhang, K. Zhao et al*.* 2009. Pyramiding *Xa23*, *Pi9* and *Bt* genes by molecular marker-assisted selection. Journal of Biology **26**:7–9. (in Chinese with English Abstract).

Duan, X. L., X. G. Li, Q. Z. Xue, M. Abo-El-Saad, D. P. Xu, and R. Wu. 1996. Transgenic rice plants harboring an introduced potato proteinase inhibitor II gene are insect resistant. Nature Biotechnology **14**:494–498.

Fang, J. 2008. The vegetative insecticidal protein genes of *Bacillus thuringiensis* and expression in transgenic rice. PhD thesis, Zhejiang Uni. Hangzhou, Zhejiang, China.

Gao, T., Y. Wu, Y. Zhang, L. Liu, Y. Ning, D. Wang, H. Tong et al*.* 2011. *OsSDIR1* overexpression greatly improves drought tolerance in transgenic rice. Plant Molecular Biology **76**:145–156.

Guo. Y., L. Zhang, G. Xiao, S. Y. Cao, D. M. Gu, W. Z. Tian, and S. Chen. Expression of betaine aldehyde dehydrogenase gene and salinity tolerance in rice transgenic plants. Science in China Series C: Life Sciences **140**:496–501. (in Chinese with English Abstract).

Huang, J. Q., M. W. Zhi, L. A. Hai, and X. Z. Yu. 2001. Agrobacterium tumefaciens-mediated transformation of rice with the spider insecticidal gene conferring resistance to leaffolder and striped stem borer. Cell Research **11**:149–155.

Huang, J., S. J. Sun, D. Q. Xu, X. Yang, Y. M. Bao, Z. F. Wang, H. J. Tang et al. 2009. Increased tolerance of rice to cold, drought and oxidative stresses mediated by the overexpression of a gene that encodes the zinc finger protein ZFP245. Biochemical and Biophysical Research Communications **389**:556–561.

Hu, H. H., M. Q. Dai, J. L. Yao, B. Z. Xiao, X. H. Li, Q. F. Zhang, and L. Z. Xiong. 2006. Overexpressing a NAM, ATAF, and CUC (NAC) transcription factor enhances drought resistance and salt tolerance in rice. Proceedings of the National Academy of Sciences **103**:987–992.

Jiang, G. C., G. Xu, J. M. Tu, X. H. Li, Y. Q. He, and Q. F. Zhang. 2004. Pyramiding of insectand disease-resistance genes into an elite indica, cytoplasm male sterile restorer line of rice, ‘Minghui 63’. Plant Breeding **123**:112–116.

Li, H. W., B. S. Zang, X. W. Deng, and X. P. Wang. 2011. Overexpression of the trehalose-6-phosphate synthase gene *OsTPS1* enhances abiotic stress tolerance in rice. Planta **234**:1007–1018.

Li, G. Y., X. P. Xu, Q. Xia, J. M. Fu, and G. Y. Sheng. 2003. Inheritance analysis and insects resistance of transgenic rice harboring *GNA*+*SBTi* double genes. Acta Scientiarum Naturalium Universitatis Sunyatseni **42**:125–126. (in Chinese with English Abstract).

Lin, X. F., Z. M. Liu, D. P. Liu, W. Y. Hao, K. X. Tang, and T. L. Wu. 2006. Introduction double insect resistance genes *cryIA* (a)-*pta* to japonica rice and assessment of resistance to rice stem borer. Molecular Plant Breeding **4**:345–350. (in Chinese with English Abstract).

Liu, Z., J. Zhao, Y. Li, W. Zhang, G. Jian, Y. Peng, and F. Qi. 2012. Nonuniform distribution pattern for differentially expressed genes of transgenic rice Huahui 1 at different developmental stages and environments. PLoS One **7**:e37078.

Liu, Q., Z. Wang, X. Chen, X. Cai, S. Tang, H. Yu, J. Zhang et al. 2003. Stable inheritance of the antisense Waxy gene in transgenic rice with reduced amylose level and improved quality. Transgenic Research **12**:71–82.

Ma, J., Y. Song, B. Wu, M. Jiang, K. Li, C. Zhu, and F. Wen. 2011. Production of transgenic rice new germplasm with strong resistance against two isolations of Rice stripe virus by RNA interference. Transgenic Research **20**:1367–1377.

Qiu, D., J. Mao, X. Yang, and H. Zeng. 2009. Expression of an elicitor-encoding gene from *Magnaporthe grisea* enhances resistance against blast disease in transgenic rice. Plant Cell Reports **28**:925–933.

Rao, X. L., X. H. Zhang, R. J. Li, H. T. Shi, and Y. T. Lu. 2011. A calcium sensor-interacting protein kinase negatively regulates salt stress tolerance in rice (*Oryza sativa*). Functional Plant Biology **38**:441–450.

Rao, K. V., K. S. Rathore, T. K. Hodges, X. Fu, E. Stoger, D. Sudhakar, W. Sarah et al. 1998. Expression of snowdrop lectin (GNA) in transgenic rice plants confers resistance to rice brown planthopper. Plant Journal **15**:469–477.

Shu, Q. Y., G. Y. Ye, H. R. Cui, X. Y. Cheng, Y. B. Xiang, D. X. Wu, M. W. Gao et al. 2000 Transgenic rice plants with a synthetic *cry1Ab* gene from *Bacillus thuringiensis* were highly resistant to eight lepidopteran rice pest species. Molecular Breeding **6**:433–439.

Song, W. Y., G. L. Wang, L. L. Chen, H. S. Kim, L. Y. Pi, T. Holsten, J. Gardner et al. 1995. A receptor kinase-like protein encoded by the rice disease resistance gene, *Xa21*. Science **270**:1804–1806.

Su, J., G. M. Chen, D. G. Tian, Z. Zhu, and F. Wang. 2008. A gene encodes 5-enolpyruvylshikimate-3-phosphate mutagenized by error-prone PCR conferred rice with high glyphosate-tolerance. Molecular Plant Breeding **6**:830–836. (in Chinese with English Abstract).

Su, J., C. Hu, X. Yan, Y. Jin, Z. Chen, Q. Guan, Y. Wang et al. 2015. Expression of barley SUSIBA2 transcription factor yields high-starch low-methane rice. Nature **523**:602–606.

Tang, W., H. Chen, C. G. Xu, X. H. Li, Y. J. Lin, and Q. F. Zhang. 2006. Development of insect resistant transgenic *indica* rice with a synthetic *cry1C** gene. Molecular Breeding **18**:1–10.

Tang, K. X., P. Tinjuangjun, Y. N. Xu, X. F. Sun, J. A. Gatehouse, P. C. Ronald, H. X. Qi et al. 1999. Particle-bombardment-mediated co-transformation of elite Chinese rice cultivars with genes conferring resistance to bacterial blight and sap-sucking insect pests. Planta **208**:552–563.

Tu, J. M., G. A. Zhang, K. Datta, C. G. Xu, Y. Q. He, Q. F. Zhang, G. S. Khush et al. 2000. Field performance of transgenic elite commercial hybrid rice expressing *Bacillus thuringiensis* delta-endotoxin. Nature Biotechnology **18**:1101–1104.

Wang, F. Z., Q. B. Wang, S. Y. Kwon, S. S. Kwak, and W. A. Su. 2005. Enhanced drought tolerance of transgenic rice plants expressing a pea manganese superoxide dismutase. Journal of Plant Physiology **162**:465–572.

Wang, Z. X., M. Yano, U. Yamanouchi, M. Iwamoto, L. Monna, H. Hayasaka, Y. Katayose et al. 1999 The *Pib* gene for rice blast resistance belongs to the nucleotide binding and leucine-rich repeat class of plant disease resistance genes. Plant Journal **19**:55–64.

Wang, A. J., F. Y. Yao, F. J. Wen, C. X. Zhu, G. X. Li, L. Yang, Q. S. Zhu et al. 2002. Obtaining of transgenic rice plants resistant to both stem borer and bacterial blight disease from *Bt* and *Xa21* genes transforming. Acta Agronomica Sinica **28**:857–860. (in Chinese with English Abstract).

Wang, Y. N., L. Zhang, Y. H. Li, L. Z. Han, Y. M. Liu, Z. Zhu, J. Su et al*.* 2014. Expression of Cry1Ab protein in a marker-free transgenic Bt rice line and its efficacy in controlling a target pest, *Chilo suppressalis* (Walker) (Lepidoptera: Crambidae). Environmental Entomology **43**:528–536.

Wu, H., H. Ye, R. Yao, T. Zhang, and L. Xiong. 2015. *OsJAZ9* acts as a transcriptional regulator in jasmonate signaling and modulates salt stress tolerance in rice. Plant Science **232**:1–12.

Xia, X., X. Fan, J. Wei, H. Feng, H. Qu, D. Xie, A. J. Miller et al. 2015. Rice nitrate transporter OsNPF2. 4 functions in low-affinity acquisition and long-distance transport. Journal of experimental botany **66**:317–331.

Xiang, Y. B., Z. Q. Liang, M. W. Gao, Q. Y. Shu, G. Y. Ye, X. Y. Cheng, and I. Altosaar. 1999. Agrobacterium-mediated transformation of insecticidal *Bacillus thuringiensis* *cry1Ab* and *cry1Ac* genes and their expression in rice. Chinese Journal of Biotechnology **15**:494–500. (in Chinese with English Abstract).

Xie, D. X., Y. L. Fan, and P. C. Ni. 1991. *Bacillus thuringiensis* insecticidal gene into cultivated rice varieties in China on the 11th to spend to obtain transgenic plants. Science in China Series B **24**:830–834. (in Chinese with English Abstract)

Xu, D. P., Q. Z. Xue, D. McElroy, Y. Mawal, V. A. Hilder, and R. Wu. 1996. Constitutive expression of a cowpea trypsin inhibitor gene, *CpTI*, in transgenic rice plants confers resistance to two major rice insect pests. Molecular Breeding **2**:167–173.

Yan, W. H., P. Wang, H. X. Chen, H. J. Zhou, Q. P. Li, C. R. Wang, Z. H. Ding et al*.* 2011. A major QTL, *Ghd8*, plays pleiotropic roles in regulating grain productivity, plant height, and heading date in rice. Molecular Plant **4**:319–330.

Yang, H., J. Li, S. Guo, X. Chen, and Y. Fan. 1989. Transgenic rice plants produced by direct uptake of δ-endotoxin protein gene from *Bacillus thuringenesis* into rice protoplasts. Scientia Agricultura Sinica **22**:1-5. (in Chinese with English Abstract).

Yao, F. Y., C. X. Zhu, G. X. Li, and F. J. Wen. 2002. Identification of *Bt* rice resistance to stripe stem borer and genetic analysis of transgenes. Scientia Agricultura Sinica **35**:142–145. (in Chinese with English Abstract).

Ye, G. Y., J. M. Tu, H. R. Cui, K. Datta, and S. K. Datta. 2001. Transgenic IR72 with fused *Bt* gene *cry1Ab*/*cry1Ac* from *Bacillus thuringiensis* is resistant against four lepidopteran species under field conditions. Plant Biotechnology **18**:125–133.

Ye, R. J., H. Q. Huang, Y. Zhou, T. Y. Chen, L. Liu, X. H. Li, H. Chen et al. 2009. Development of insect-resistant transgenic rice with *Cry1C**-free endosperm. Pest Management Science **65**:1015–1020.

Ye, X. D., S. Al-Babili, A. Kloti, J. Zhang, P. Lucca, P. Beyer, I. Potrykus et al. 2000. Engineering the provitamin A (beta-carotene) biosynthetic pathway into (carotenoid-free) rice endosperm. Science **287**:303–305.

Zeng, Q. C., Q. Wu, K. D. Zhou, D. J. Feng, F. Wang, J. Su, I. Altosaar et al. 2002. Obtaining stem borer-resistant homozygous transgenic lines of Minghui 81 harboring novel *cry1Ac* gene via particle bombardment. Acta Genetica Sinica **29**:519–524. (in Chinese with English Abstract).

Zha, X., X. Luo, X. Qian, G. He, M. Yang, Y. Li, and J. Yang. 2009. Over-expression of the rice *LRK1* gene improves quantitative yield components. Plant Biotechnology Journal **7**:611–620.

Zhang, B. J., L. L. Ling, R. F. Wang, and D. M. Jiao. 2009. Photosynthetic characteristics and effect of ATP in transgenic rice with phosphoenolpyruvate carboxylase and pyruvate orthophosphate dikinase genes. Photosynthetica **47**:133–136.

Zhang, C., Y. Song, F. Jiang, G. Li, Y. Jiang, C. Zhu, and F. Wen. 2012. Virus resistance obtained in transgenic tobacco and rice by RNA interference using promoters with distinct activity. Biologia Plantarum **56**:742–748.

Zhang, H., W. Liu, L. Wan, F. Li, L. Dai, D. Li, Z. Zhang et al. 2010. Functional analyses of ethylene response factor JERF3 with the aim of improving tolerance to drought and osmotic stress in transgenic rice. Transgenic Research **19**:809–818.

Zhang, L., S. Xiao, W. Li, W. Feng, J. Li, Z. Wu, X. Gao et al*.* 2011. Overexpression of a Harpin-encoding gene *hrf1* in rice enhances drought tolerance. Journal of Experimental Botany **62**:4229–4238.

Zhao, F. Y., Z. L. Wang, Q. Zhang, Y. X. Zhao, and H. Zhang. 2006. Analysis of the physiological mechanism of salt-tolerant transgenic rice carrying a vacuolar Na+/H+ antiporter gene from *Suaeda salsa*. Journal of Plant Research **119**:95–104.

Zhao, H. Y., Y. J. Zhang, K. M. Wu, K. J. Zhao, Y. F. Peng, and Y. Y. Gu. 2004. Expression of *Cry1Ac*/*CPTI* transgenic rice and its resistance in different stages to *Chilo suppressalis*. Journal of Agricultural Biotechnology **12**:76-79.

Zhou, M., H. L. Xu., X. L. Wei., Z. Q. Ye., L. P. Wei., W. M. Gong, Y. Q. Wang et al. 2006. Identification of a glyphosate-resistant mutant of rice 5-enolpyruvylshikimate 3-phosphate synthase using a directed evolution strategy. Plant Physiology **140**:184–195.

Zhu, C. X., A. Q. Hu, F. J. Wen, C. C. Zheng, and J. Zhang. 1999. Production of insect-resistant rice plants transformed with *cry1Ab* and *pinⅡ* genes. Journal of Agricultural Biotechnology **7**:259–266.
